# Participation of adults with cognitive, physical, or psychiatric impairments in family of origin and intimate relationships: a grounded theory study

**DOI:** 10.1186/s12889-020-08770-x

**Published:** 2020-05-07

**Authors:** Andreas Pfister, Pia Georgi-Tscherry, Fabian Berger, Michaela Studer

**Affiliations:** 1 Lucerne University of Applied Sciences and Arts, School of Social Work, Institute of Social Management, Social Policy and Prevention, Werftestrasse 1, Postfach 2945, CH-6002 Lucerne, Switzerland; 2 Lucerne University of Applied Sciences and Arts, School of Social Work, Institute of Social Pedagogy and Education, Werftestrasse 1, Postfach 2945, CH-6002 Lucerne, Switzerland; 3 Careum School of Health, Careum Research, Pestalozzistrasse 3, CH-8032 Zurich, Switzerland; 4 University of Applied Sciences in Special Needs Education, Schaffhauserstrasse 239, Postfach 5850, CH-8050 Zurich, Switzerland

**Keywords:** Participation, Family of origin, Relationship, Cognitive impairment, Physical impairment, Psychiatric impairment, Facilitators, Barriers, Grounded theory, Disability

## Abstract

**Background:**

How adults with disabilities perceive participation has received little attention. Our purpose was to formulate a grounded theory on participation, based on the subjective experience of adults with cognitive, physical, or psychiatric impairment(s), and to identify barriers, facilitators, and support needs concerning participation in different areas of life. We aimed to explore whether the United Nations’ Convention on the Rights of Persons with Disabilities (CRPD), ratified by Switzerland in 2014, and its principles are being met. Here we report on the main category and focus on the participation areas ‘family of origin’ and ‘intimate relationships.’

**Methods:**

In a qualitative, grounded theory study, we conducted problem-centered interviews with 23 adults with cognitive, physical, or psychiatric impairments (30–53 years; 11 men, 12 women), with different housing (on their own, assisted living, with parents) and work situations (primary vs. secondary labor market) in nine German-speaking Swiss cantons.

**Results:**

Participation can be understood as a continuum that extends on a horizontal level (from participation is restricted to participation takes place) and a vertical level (separative setting vs. inclusive setting). In separative as well as in inclusive settings, diverse levels of participation are possible. Many participants were stuck in an ‘in-between’ area between separative and inclusion-oriented settings. In the family of origin, there was a thin line between fulfilling relations that enhance participation and conflictual relations and overprotective parenting that limit participation. In intimate relationships, opportunities for participation were limited overall. Many interviewees were single. Social environment and family of origin (e.g., parents) can enable and facilitate intimate relationships and sexual contacts but can also be an important barrier.

**Conclusions:**

Participation can be understood as a continuum. Participation restrictions exist in separative as well as in inclusive-oriented settings, also in the areas of family of origin and intimate relationships. Participation barriers must be torn down in separative as well as in inclusion-oriented settings. Trajectories to inclusive settings should be facilitated. Families with children with impairment(s) should be supported from early on to create the best possible participation possibilities for the (adult) person with impairment(s) and to support the family of origin itself.

## Background

Switzerland ratified the United Nations’ Convention on the Rights of Persons with Disabilities (CRPD) in 2014. In the CRPD, “full and effective participation and inclusion in society” (Article 3) is a central principle to be met by the contracting nations [1]. For this reason, Pro Infirmis, one of the largest organizations in Switzerland for persons with disabilities, wanted to know how persons with disabilities in the German-speaking part of Switzerland participate in society and how they subjectively experience participation in their daily life (barriers, facilitators, etc.). The overall aim of this study was to formulate a grounded theory on participation, based on the subjective perspective of adults with different impairments (cognitive, physical, psychiatric), in order to provide insights into the complex processes of participation in various areas of life.

The World Health Organization (WHO) defines participation in the *International Classification of Functioning, Disability and Health* (ICF) as “involvement in a life situation” and defines participation restrictions as “problems an individual may experience in involvement in life situations” [2] . However, the definition of participation is not uniformly clarified [3]. The term often remains nebulous and functions as a “container term” for a multitude of different approaches and concepts [4]. Schmidt and Dworschak [5] emphasize the inherent relativity of the term: Participation is interpreted by the social environment and society based on norms and standards; at the same time, individuals are a part of this environment, and they assess participation on the basis of their personal, subjectively shaped perceptions.

This inherent relativity is somehow also part of the WHO’s biopsychosocial model of disability. Whether participation is restricted or not depends on the outcomes and interactions between health conditions (diseases, disorders, and injuries) and contextual factors (environmental and personal factors) [2]. The verb “experience” in the WHO definition of “participation restrictions” tells us that the state of participation can never be judged and understood solely from an ecological, social, or even expert perspective but is always experienced individually on its own, as an act of subjectively shaped perception by each individual human being.

Taking into account the importance of the subjective experience of participation of persons with impairment(s), it is striking that in the social sciences this aspect has received little attention up to now. A few publications are available for Switzerland and Germany, for example by Parpan-Blaser et al., Meier, and Seifert [6–8]. Recent international research on the phenomenon of participation, using qualitative methods in social research such as a grounded theory approach, have focused most often on a certain life area of participation as well as on a particular type of impairment [9–14]. No current studies have reconstructed the phenomenon of participation in a holistic way, across various forms of impairment and areas of life, and against the background of the subjective experience and assessment of participation by people with impairments. The present study aimed at closing this research gap. We put forward the following research questions:

- What possibilities and restrictions concerning participation affect persons aged 30 to 50 years with physical, cognitive, and/or psychiatric impairments in the contexts of work, housing, education, family, relationships, and leisure time?
- What barriers and facilitators concerning participation are there, and how do persons with physical, cognitive, and/or psychiatric impairments deal with them?
- What resources can persons with physical, cognitive, and/or psychiatric impairments access themselves? What kind of support is needed?

Partial results of the study were published in German, focusing exclusively on the main category “participation as a continuum” [15] and participation in the area of work [16]. To deepen our understanding of the qualitatively reconstructed phenomenon of participation, in this study we wanted to take a closer look at participation in romantic/sexual relationships and in family of origin. A Dutch cohort study stated that only 26% of young adults with cerebral palsy had a current relationship, compared to 63% in the reference sample of Dutch students [17]. Participation restrictions in the area of romantic/sexual relationships are still prevalent. What this means in daily life for persons with impairments and how participation (restriction) is experienced subjectively in this area has not yet been explored sufficiently in current research. The importance of the family of origin and social networks for participation in other areas of life (e.g., work) has been documented scientifically [14, 18]. In a Canadian sample using a grounded theory approach, Petner-Arrey et al. provide insights on the role that parents play in facilitating employment opportunities for adults with intellectual and developmental disability [14]; parents thought about what job (opportunities) fitted their adult child best, helped their child with job application processes, and provided on-the-job assistance (e.g., when problems or questions emerged). Although very instructive, what is not mentioned in Petner-Arrey et al. is what participation possibilities exist for adult persons with disability in the family of origin itself. How fulfilling and close are their relations with their family of origin? What are the barriers to participation in the family of origin? Are parents predominantly a source of help when it comes to participation in society, or can they also be a barrier? Even though these questions are highly relevant for policy, practice, and research, current research provides only insufficient answers.

This article is structured as follows: After describing how the study was conducted in the methods section, we briefly introduce the main category ‘participation as a continuum’ that we constructed from the verbal data. We then focus on barriers and facilitators of participation in the contexts of family and sexual/intimate relationships. In the discussion section, we outline implications of the findings and limitations of the study. We conclude by pinpointing the relevance of the study results for future policy, practice, and research.

## Methods

This study was carried out with qualitative-interpretive methods by using grounded theory [19]. We thought that this discovering and hypothesis-generating method would be very appropriate for fathoming the social participation of people with disabilities in various areas of life as openly and as in depth as possible and for elaborating the concepts and dimensions found empirically in the best possible way. As demonstrated in other grounded theory studies, this methodology can produce highly relevant and innovative research outcomes for policy and practice, especially when it comes to underexplored and socially marginalized or excluded population groups [14, 20–22]. A completed COREQ checklist can be found in Additional file 1.

### Data collection

Drawing on grounded theory [19], we gathered and analyzed the data in an iterative and cyclic way. The phases of data collection and evaluation alternated. Data was collected from December 2015 to October 2016 in the German-speaking part of Switzerland.

#### Inclusion criteria and sampling strategy

A theoretical sampling strategy was applied [19]. Through a progressive and targeted selection of persons, situations, and events, which contribute to the further development and saturation of a grounded theory on participation of persons with disabilities, it was ensured that the empirical material could be understood in all its complexity [23].

Due to the nature of commissioned research, besides the theoretical sampling strategy, Pro Infirmis, which funded the study, defined general inclusion criteria for the study in a collaborative process with the research team. Persons that met the following criteria were included:

- Aged 30–50 years
- Residing in the cantons of Basel-Stadt/Basel-Land, the Grisons, Lucerne, Nidwalden, Obwalden, Schaffhausen, Thurgau, or Zurich (all in the German-speaking part of Switzerland)
- With cognitive or/and physical or/and psychological impairments

The study focused on 30- to 50-year-olds, since in this age group we can expect to find participation—from a normative point of view—in all areas of life investigated (e.g., due to completed education/initial training, sexual development). The defined age range provided an optimal basis for exploring participation facilitators and restrictions. We aimed to include a minimum of one to two persons per the cantons mentioned above, as Pro Infirmis has offices in these cantons that contributed financially to the study. The wide spectrum of impairments was chosen explicitly to be able to reconstruct the phenomenon of participation in a holistic way rather than focused on or limited to a certain type of impairment.

#### Recruitment of participants

The study participants were recruited via a database from the University of Applied Sciences in Special Needs Education in Zurich. The database stemmed from a former research project [6]. Further, we contacted foundations, leisure clubs, sports groups for people with disabilities, etc., and cantonal offices and expert agencies that are in contact with persons with disabilities. The respective persons in charge were asked to hang up posters, hand out flyers, and ask people with impairments personally if they would participate. Possible study participants or their representatives (close family members, directly involved care persons, social workers, etc.) that contacted the research team (by e-mail, phone) were asked for age, gender, civil status, residential municipality, housing type, nationality, highest education, occupation, form of impairment, children, and main source of income. This data was the basis for determining, following the general inclusion criteria and the theoretical sampling strategy, whether a person was included in the study or not.

#### Problem-centered interviewing

Verbal data was collected with the instrument of the problem-centered interview [24, 25], which is a loosely structured in-depth interview. This approach is also adequate for people with cognitive impairment [26]. A version of the interview guide was constructed in easy language. Participants were free to choose the interview location (e.g., at home, at the university). Prior to the interview, researchers informed the interviewees about the study (goals, data handling, etc.). Participants, or in case of severe impairments their legal representatives (e.g., parents), gave oral and written consent. The interviews were recorded (audiotaped); the duration of the interviews ranged from 30 min to 3 h, with an average duration of 90 min. The interview guide ([27], Appendix C) was developped for the overall study that included all participation areas. Drawing on the form of a problem-centered interview, an opening question was posed at the beginning: “I’m interested in how you live. What does your everyday life look like? Where can you participate, make decisions, and where is it sometimes difficult? Please just tell me about this.” This opening question enabled free narratives, on the basis of which the interviewer asked immanent (e.g., “Can you tell me more about that?”) and exmanent questions (e.g., “You told me about your mother supporting you in daily life? What about your father?”). We then asked about intimate relationships and family of origin in order to further explore barriers and facilitators for participation in those areas. At the end of the interview, sociodemographic characteristics and more narrowly defined themes (such as association memberships) were gathered using a standardized short interview questionnaire. We assessed the type of impairment by asking “How would you describe your impairment?” and complemented the answers with our own perspective, based on the ICF framework [2]. We then categorized the type of impairment as *cognitive* (e.g., intellectual impairments, learning disabilities, traumatic brain injuries), *physical* (e.g., para- and tetraplegic, multiple sclerosis, neurodegenerative diseases), or *psychiatric* (e.g., depression; anxiety, obsessive-compulsive, and bipolar disorders). A clear demarcation of the form of impairment was not always possible. Several persons named multiple types of impairment, e.g., psychiatric and physical impairments. These persons were assigned to the type of impairment that affected them the most. After completing the interview, we noted down the circumstances of the interview (e.g., setting of the interview, interactions between interviewer and interviewee) in a postscript.

### Participant characteristics

The final sample consisted of 23 persons, 12 women and 11 men (see participant characteristics in Table 1). Despite the age limit of 50 years, two persons above the age range (a 51- and a 53-year-old) were included in the final sample, due to theoretical sampling reasons and the absence of suitable participants fitting the age range.

**Table 1:** Study sample (*n* = 23)

	Total
**Gender**	
Female	12
Male	11
**Age in years**	
30–35	10
36–40	3
41–45	3
46–50	5
51–53	2
**Marital status**	
Unmarried	19
Married or in registered same-sex partnership	3
Divorced	1
**Workplace**	
Primary labor market ^a^	3
Reinsertion measure into primary labor market	1
Secondary labor market ^a^	12
Disability insurance pension (without working)	5
Non distinctive	2
**Type of impairment**	
Physical	6
Cognitive	11
Psychiatric	6
**Housing**	
On their own	6
With a partner	3
On their own (with personal assistance)	2
Assisted living / residential care (external living unit)	6
Own apartment with relatives	1
With parents / in the family of origin	5
**Canton**	
Basel-Land	2
The Grisons	2
Lucerne	6
Nidwalden	1
Obwalden	2
Schaffhausen	4
Solothurn	1
Thurgau	2
Zurich	3

^a^ The primary labor market is the regular labor market. In the secondary labor market, employees receive no wages or reduced regular wages, because they are temporarily or constantly unemployed. Specialized firms receive money to provide appropriate jobs. These secondary labor market programs aim, for example, at vocational integration. Or they provide a longer-term daily structure for people with very few employment opportunities in the primary labor market (e.g., people with severe disabilities)

### Data analysis

Audio files were fully transcribed and anonymized (names, places, etc.). The audio files and transcripts were then uploaded into the qualitative data analysis software MAXQDA and stored safely on a Swiss university server. Verbal data was coded using techniques and procedures described by Strauss and Corbin [19].

First, using open coding strategy, the verbal data was analyzed and coded along the main research questions and the areas of life. We validated the analysis communicatively. A second researcher looked over the analysis and discussed and adjusted differences in codings and codes with the primary researcher.

Second, based on the open coding, categories and their dimensions and specifications were built. We then moved to axial coding and formulated the categories at a higher level of abstraction and formed code axes.

Third, not yet analyzed interview transcripts were coded with the code axes formed (axial and selective coding). As suggested by grounded theory, analysts oscillated between deductive and inductive analysis. Existing categories were tested, reviewed, enriched, and further elaborated using new interview data. We also tested for data saturation by analyzing whether new aspects emerged or code axes and concepts were confirmed by new interview data.

The last step in the analysis was to form a core category (‘participation as a continuum’; see results section below) that best captured and summarized the empirical phenomenon. The results were discussed and validated with the Pro Infirmis steering committee for the study; the members of the steering committee were disability experts and heads of cantonal Pro Infirmis offices. The aim was to discover potential blind spots in the analysis and to ensure scientifically sound results.

## Results

### Participation as a continuum

**Fig. 1:**
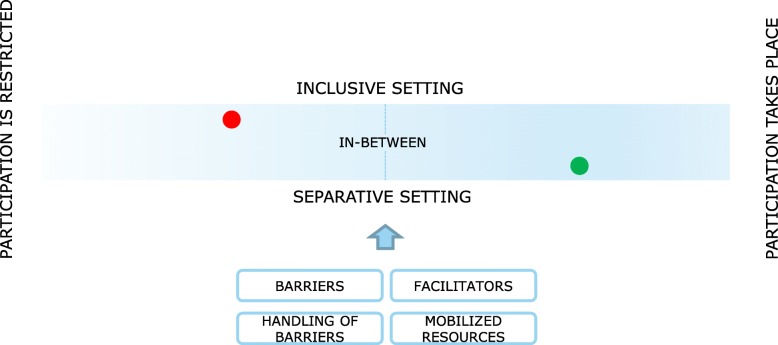
Participation as a continuum

Following the narratives of the interviewees, participation can be understood as a continuum (see Fig. 1). The interviewees’ subjective experience of participation in the areas of housing, education, work, family, relationship, and leisure time can be located along this continuum.

The participation continuum extends on a horizontal level (from participation is restricted to participation takes place) and a vertical level (separative setting vs. inclusive setting). Inclusive settings cannot be equated with complete participation, and separative settings cannot be equated with participation that is always restricted. When we consider the interviewees’ subjective view, in general all levels of participation are possible in separative as well as in inclusive settings. For example, some interviewed persons living in an ‘inclusion-oriented setting’ lived on their own in an apartment where they were totally independent. Nevertheless, some of them faced diverse barriers (e.g., restricted social contacts). Due to these barriers, they concluded that their participation was restricted (see red point in Fig. 1). On the other hand, we identified persons that lived in a highly separative setting, e.g., in a residential unit for persons with intellectual impairments, and not all of those interviewees were dissatisfied with their current housing situation. Some were satisfied and perceived participation possibilities (see green point in Fig. 1). They were not currently considering a change to a more inclusive setting. Such a change would even be a burden at the present time, as they explained in the interviews (see example below).

*A2: “Unfortunately, I have to say that yesterday the team* [the carers, social workers] *did something that I do not like. Behind my back they organized an own apartment for me. ( … .). I wish that I can simply be in a residential group with other people and have my peace. That I just have to cook and clean the kitchen. And that the carers don’t put me under so much pressure. That is what I want*” (A2, male, age 34, cognitive impairment).

In family of origin and relationships as well, inclusive settings cannot be equated with complete participation, and separative settings cannot be equated with participation that is always restricted. For example, some interviewees living in their parent’s home in a quite separated context perceived their participation possibilities as good overall. Others, living in the same setting, saw them as restricted. They experienced limited space for self-determined decision-making and sexual activities due to overprotective parenting by their parent(s) (see more in detail in the paragraphs below).

Even though in general all levels of participation are possible in separative as well as in inclusive settings, a significant part of the interviewees wanted to move towards more inclusion-oriented settings. But they were currently facing various barriers that made it impossible to live in an inclusion-oriented setting. They were stuck in a kind of ‘in-between’ between separative and inclusive settings. For example, some of the respondents did not have any opportunity to pursue gainful employment in the primary labor market, but at the same time they were not challenged enough in the structures of the secondary labor market. It was impossible to contribute and develop themselves in the secondary labor market either personally or professionally. And, due to selectivity and structures of the primary labor market, it was impossible for them to move towards primary labor market employment.

Barriers, facilitators, the handling of barriers, and the mobilization of resources had an impact in every area of life on where persons with disabilities stand on the ‘participation continuum’ and on what kind of support was needed (see Fig. 1). This was also the case in the context of family of origin and relationship.

### Participation in the family of origin

According to the narratives and the understanding of the interviewees, the family of origin includes parents, siblings, and grandparents. Not all of them were mentioned in each interview. The interviewees prioritized certain family members, due to their relevance and importance in relation to participation, as being a special source of support or an important barrier to participation. Qualitative data material showed that there was a thin line between supportive/caring and overprotective families of origin. Overprotective families potentially limit participation.

#### Participation takes place; facilitators

The data showed that if there were good social and caring relationships between interviewees and their family of origin, the members of the family of origin were often important reference and support persons for a person with an impairment; they facilitate participation. The support provided by the family of origin was very diverse.

Despite being an adult person, some interviewees—especially persons with cognitive impairment—lived in their parent’s home. There are several reasons for this. For one, the interviewees said that they had and got everything they needed at their parent’s home. For another, parents can cushion the interviewees’ financial burden, since living in an (external) residential group can be very expensive. Even if interviewees no longer lived together with their parents, some of them had daily contact with their parents: *“I actually go to my parents’ every day for lunch and dinner”* (B6, male, 35 years, physical impairment).

Some interviewees mentioned that their parents had offered substantial guidance concerning the choice of appropriate vocational training when interviewees were at a younger age. Even nowadays—at the time of the interview—these parents seemed to help and support their son or daughter when they faced problems at work or in housing.

Parents also provided help concerning the financial literacy of their son/daughter with cognitive impairment or handled organizational tasks, e.g., by keeping in contact and managing relations with hospitals, doctors, and so on. The following quote demonstrates again that for some interviewees, parents played an important role in supporting them and fighting for their needs: *“She contacts the university hospital when something happens. My parents are very important to me, because I could not defend myself alone”* (A2, male, age 34, cognitive impairment).

In addition to parents, some siblings also provided support for the interviewees. As the data showed, this support was mostly in the area of leisure time activities or holiday arrangements (e.g., creating opportunities to spend holidays together).

For persons with psychiatric impairments, an important facilitator of participation in the family of origin was family awareness of the family’s history of illness(es). As the data showed, the interviewees experienced more understanding for their situation if certain symptoms were already prevalent and were known and accepted within the family of origin. This seems to create a safe space and enhances interviewees’ capacity to share their experiences and concerns (related to psychiatric impairments) with their family of origin: *“My great-grandmother has already had depression, my grandmother and my mother, and me, too. So I am the fourth generation. My father also grew older by being surrounded by that. He knew it in my mother-in-law and from my mother, and … Yes, we know that about each other, and we talk about it, and … you can feel it, too*” (B4, female, age 50, psychiatric impairment).

From the verbal data we found that persons with cognitive impairment had a general tendency to receive more support and attention from parents and siblings than persons with psychiatric and/or physical impairments, even though also for persons with psychiatric and physical impairments their family of origin was important and could play a supportive role (as demonstrated above).

#### Participation is restricted; barriers

In addition to interviewees who assessed their relationship with the family of origin positively, there were also persons who reported a lack of relationships or very difficult relationships. Especially persons with psychiatric impairment experienced difficult familial relationships and reported a lack of support from their family of origin. Some of these interviewees stated that their problems or wishes were often ignored or denied by the family of origin: “*I have never actually had a place of retreat in the way that I could go to my parents if I had a problem ...*” (B2, female, age 32, psychiatric impairment).

The family of origin, especially parents of interviewees with cognitive impairment, sometimes found it difficult to reduce their support or to recognize when the interviewees were able to carry out certain actions independently, as the data showed. This became evident in everyday actions such as getting dressed:

*A12: “In the outdoor residential group I could make the clothes ready myself and dress as I wanted, and now that I am in the ‘mummy and daddy hotel,’ mummy puts out my clothes for me again.*

*I: Ah yes?*

*A12: Yes.*

*I: But you could say: ‘I'll do it myself.’*

*A12: Yes, I could. But then she says again: No, it doesn’t go together”* (A12, female, age 36 years, cognitive impairment).

Some parents and siblings make important decisions concerning housing, work, relationships, and contraception. These decisions can limit the self-determination and participation of the person with an impairment. For example, a 43-year-old woman with cognitive impairment said in the interview that her brother placed restrictions on a new housing arrangement. The same woman also perceived restrictions coming from her parents concerning having a love relationship including sexual activities with her boyfriend: “*I’ve brought a boyfriend to my house before. But the parents didn’t like him. ... And then I wanted to have... Then the parents came and said: ‘Yes, you may not do that. I have a contraceptive here’*” (A11, female, age 43, cognitive impairment). Especially persons with a cognitive impairment often seemed to be dependent on their parents’ consent regarding their love relationships and sexual activity. Further, the data showed that especially women with a cognitive impairment are influenced by their parents in matters of pregnancy prevention and even sterilization:

*A12: “Because I love children, but I will never know for myself what it is like to have children.*

*I: Why do you say that?*

*A12: Because I had to be sterilized.*

*I: Why did you have to do that?*

*A12: Because mum and dad say: If I have children, they would have to take care of them anyway and raise the children. I could not do that*” (A12, female, age 36, cognitive impairment).

#### Handling of barriers

What happens when conflicts, bad relations with the family of origin, or overprotective behavior of parents hinders the well-being and participation of persons with impairments in the family of origin? How did the interviewees handle these barriers and cope with the situation?

As a possible coping strategy, some of the interviewees withdrew or completely broke off contact with the family: “*And I just had to say, uh, it tears me apart every time I talk to him* [father] *on the phone or hear from him. These memories come up again and again. And that also brings me down into the hole. And I just had to say: ‘Uh, no, I don’t want any more.’ And that is now about ... 4 to 5 years. Yes. And I don’t want to stay in contact with him anymore. Not with mother anyway ...*” (B5, female, age 39, psychiatric impairment).

Other respondents forgave individual family members and approached them again gradually: “*I’m getting closer to my family again. .... Today I have to say ... Something went wrong ..., but I can forgive my parents again*” (B2, female, age 32, psychiatric impairment).

Interviewed persons with cognitive impairment that were confronted with overprotective behavior on the part of their family of origin (mostly by their parents) reacted basically with two coping strategies. Some distanced and detached themselves from their parents. They made it clear to their parents that they wanted to act independently. This could be very emotional for both parties: “*For example, she accompanied me to the training location. In the beginning, I was happy, but after a few weeks I said: ‘Mommy, I don’t want you to come with me!’ I was talking to a wall, and then I just said: ‘Mother, go away!’ And then she cried, but so did I.*” (B1, female, age 31, cognitive impairment). Other interviewees became accustomed to given circumstances and accepted them, even when solutions preferred by their parents were not very attractive for them: “*And then mum and dad showed me that and said: ‘How about we take you down to C-Street? Then you could live with us at home’ (....) That’s when I first freaked out. (....) And now I’m at home and living with my parents. (...) And, yes. I have it actually good.*” (A12, female, age 36, cognitive impairment). These types of statements, which were also found for other areas of participation in the verbal data, were coded as coping strategies of acceptance and reframing.

#### Support needs

Interestingly, interviewees did not mention professionals at all, such as social workers, physicians, nurses, or psychologists, who could have helped them manage their relationship with the family of origin. It seems that the individuals and the families had to deal with their relationships by themselves. The verbal data revealed a lack of professional family counseling and support from an early age on.

### Participation in the context of a relationship

The verbal data provided reports on life in a relationship, as a single person, as a widow, as a divorcee, life in a marriage or registered same-sex partnership, life in a long-distance relationship, life as parents, and life as a childless person. The interviewees reported various types of relationship status, but at the time of the interview the majority of the interviewees lived on their own as single persons.

#### Participation takes place; facilitators

Life as a single person was accepted by some of the interviewees in a self-determined way, at least for the time being. These interviewees were not actively seeking a boyfriend or girlfriend. They named different reasons for being single: Some persons wanted to wait until the “right person” appeared. Others did not yet feel ready for a new relationship due to the death of or separation from the most recent partner. Some interviewees emphasized that other aspects in their life, such as individual career development, were currently more important to them than having a relationship.

Even though some persons self-identified as voluntary singles, a greater portion of the interviewees had a preference for a relationship. One man with physical impairments stressed the importance for him of having experienced a long-term love relationship with a woman as follows: “*That gave me very, very much. At all uh ... just that someone can love me, that is an experience that* [breathes in deeply] *... yes, I unfortunately have to say... many disabled ... never ... experience.* [speaks slowly] *I just know from myself what that, what that uh... causes in self-understanding in, in* [seeks words]*. Yes, I, I would not be myself if I had not been allowed to have this experience ...*” (B8, male, age 51, physical impairment).

The interviewees mentioned the following aspects of a fulfilling and loving relationship: reciprocity, mutual trust, talking together, finding solutions together, and shared activities. Several persons with psychiatric impairments lived together with a partner who also had a psychiatric impairment. Those interviewees stressed that the insider knowledge about their partner with an impairment was a facilitator for the relationship. It ensured that there was a good basis for understanding the interviewee’s daily problems. Psychiatric care was sometimes even the starting point of a relationship: “*I finally met my boyfriend in psychiatric care. … That is of course an advantage, because he knows this condition and got to know me under these circumstances. So, he cannot come now and say* [speaks with a deeper voice]*: ‘Yeah, I didn’t know that’ or ‘I didn’t know how bad that could be’ or whatever. And um, yes, I get a lot of understanding and support from him*” (B4, female, age 50, psychiatric impairment). Further, for persons with cognitive impairment, the social environment could serve as a substantial facilitator for self-responsible and independent sexuality:

*A11:* “*There’s something nice about being in love, you know. I like it when you pet me, and kiss me, and massage, or something else. Or, I like to sleep with my boyfriend, for example, and having sex. Then, make massages.*

*I: But you can’t do that if you don’t live together at all, can you?*

*A11: Well, in the evening, mum and me … So if I invite him home, then I will*” (A11, female, age 43, cognitive impairment).

Interviewees brought up different places and opportunities to get to know potential partners besides the already-mentioned psychiatric care setting (for persons with psychiatric impairments). For example, the interviewees mentioned apprenticeships or workplace as good opportunities for making contacts and getting to know future partners. Also mentioned as possible contact options were the Internet or bars and restaurants, where they could flirt with possible partners.

#### Participation is restricted; barriers

Many interviewees reported that building a love relationship, living in a relationship, and fulfilling their sexual needs was challenging in everyday life. They viewed their participation in the life area of relationships as restricted.

Some interviewees were currently not living in a relationship, but they desperately longed for one. They were involuntarily single and identified several barriers causing them to be in that position: their own impairment, their perceived low value on the partner market, and lack of trust in other people.

Some interviewees thought that their cognitive, physical, or/and psychiatric impairment was an important reason for being single. Disabilities create strong barriers and disadvantages when it comes to relationships. Several persons put this argument forward by situating themselves in a partner market that is structured by the rules of supply and demand. In this market, visual appearance (beauty, physical appeal) and social standing (good education, integration in the labor market) are goods of great importance. They are decisive for an individual’s chances to choose or to be chosen as a potential love or sex partner. Due to their impairment(s), some interviewees thought and experienced that they could not keep up with some or all of the high expectations in this market.

A further barrier was identified on the individual level: a lack of trust in other people due to former negative socialization and biographical experiences. This made it difficult for the interviewees to enter into contact with people and build up a relationship.

Parenthood was a relevant topic in the interviews. Only a few interviewees reported that they had children of their own. Many of them had thought about being parents but decided against it with their partner. For instance, one young man reported that next to his work he had no time and energy for children. As a further argument, children were often costly. Some women with cognitive impairment stated that they had decided not to have children for health reasons. The argumentation of the interviewees with cognitive impairment in the sample suggested that the views of the close social environment (e.g., the family of origin) played a role in the decision not to have children, as was mentioned above.

Full participation in the area of relationships would also imply having sufficient possibilities for and access to sexual activities within a relationship or as a single person (e.g., casual sexual contacts). For the majority of the interviewees, this is not the case. Sexuality was a taboo subject from a social, family, and personal point of view. The interviewees criticized that their sexual needs were not being recognized by society and important reference persons (e.g., family of origin, personal assistants). Even within their circle of friends, some interviewees found that sexuality was considered embarrassing and not discussed. As one interviewee put forward, persons with impairment were often constructed in public and the media discourse as non-sexual subjects with no sexual needs: “*But I just think that this is something that public perception misses out on, so to speak: ‘Yes, disabled people, they don’t even have these needs, they don’t need that at all’. ‘They don’t need sexuality and stuff like that.’ Yes, that’s right, that’s also a lot of things that are simply very, very much in people’s minds. Also, how about disabled people is reported in the media ...how this is perceived*” (B12, male, age 35, psychiatric impairment).

Due to the difficulty of finding a sexual partner or/and a relationship, some interviewees considered professional sex workers as an option for fulfilling their sexual needs. Those interviewees mentioned sex workers in general but also specialized sex workers who specifically meet the sexual needs of persons with physical and/or cognitive impairment(s), called *Berührer/innen* (‘touchers’) in German. But neither one seemed to be a valuable alternative for the interviewees, as they would not meet their actual needs: One male interviewee with a physical impairment stated that specialized sex workers would not be able to meet his needs, due to his perception that they would just caress and cuddle him without sexual intercourse. Another man with a psychiatric impairment, after considering visiting a female sex worker, came to the conclusion that he “only” wanted to experience physical closeness such as cuddling and being hugged.

#### Handling of barriers

The interviewees reported using different coping strategies when confronted with barriers. Some persons used non-functional coping strategies that did not improve and sometimes even worsened their situation. They gave up hope of finding a partner in the near future. They then increasingly withdrew, behaved more passively in the search for a partner, or even avoided places where they could get to know potential partners.

Also in the life area of a partnership, it became apparent that some interviewees mainly focused on personal factors when they tried to identify barriers that hinder participation. Social, material, and structural factors were neglected, and mainly self-stigmatization occurred. The interviewees thought that their personal and physical attributes caused by the impairment were mostly responsible for hindered participation in the life area of a partnership.

Some persons who were living as singles tried to find positive aspects in this status, even though it became clear during the interview that they would have preferred to live together with a partner in a relationship. This coping strategy—which we also identified in other participation areas of life—was coded as ‘reframing.’

A further coping strategy was ‘looking for support.’ Persons with an impairment realized that their chances of finding a relationship were currently poor. For help, they involved their social network; for example, with financial support from their family of origin, they registered with a specialized dating agency for people with impairments.

Last but not least, the verbal data revealed diverse self-determined strategies that the interviewees implemented—without any help from other persons—to overcome or weaken participation barriers in the life area of relationships and sexuality. Some interviewees reported that they did not have any opportunities to have sex with a partner and engaged in self-simulation and masturbation: “*Because I need that, too. That’s why I do it in the evening, at home, myself*” (A12, female, age 36, cognitive impairment). One man with physical impairment gave straightforward advice to his personal assistant on how she should behave in the morning when he is in bed with his girlfriend to ensure personal and sexual intimacy.

#### Support needs

In the area of relationships and sexuality, the social environment, such as the family of origin, plays a crucial role in facilitating or hindering participation. This is especially the case for persons with cognitive impairment, as the data showed. Some barriers could be removed by sensitizing the social environment to the sexual needs of people with disabilities and by creating need-oriented family counseling and support services. Whereas the need for support mentioned above was reconstructed from the verbal data through qualitative-interpretative analysis, in the interviews the interviewees directly identified a personal support need that is of major concern to them. Although there are potentially many ways to find partners (see the section “Participation takes place; facilitators” above), it was very difficult for many of the interviewees to get to know someone. Therefore, the interviewees called for more meeting opportunities or ‘marriage markets’ like singles events and meetups that are inclusive and welcoming especially for people with impairment(s).

## Discussion

Let us first put the results into the bigger context of the overall study [27], in which we also looked at the participation areas work, housing, education, and leisure time. As in the areas of family of origin and intimate relationships, in these areas we also found facilitators and barriers for participation. Even though barriers and facilitators varied between areas, one main and strong participation barrier was found in all areas: financial deprivation. Many of the interviewees had limited financial resources [15, 27]. Analyses by the Swiss Federal Statistical Office also show that people with disabilities are at greater risk of poverty than people without disabilities [28]. Financial deprivation strongly affects participation possibilities. Persons with fewer financial opportunities, for example, had less freedom to move out of their parent’s home than persons with better financial resources. This limited not only their housing options but also participation opportunities in family of origin and intimate relationships. Further, the main results demonstrate that participation is restricted in all areas and settings, in inclusion-oriented as well as in separative settings. Many interviewees were stuck somewhere in-between separative and inclusion-oriented settings. They wanted to move towards more inclusion-oriented settings. “Full and effective participation and inclusion in society” (CRPD, Article 3) [1] is not yet the case for persons with impairments in Switzerland. This was also the conclusion of a report to the United Nations by Inclusion Handicap, the Swiss umbrella association of disability organizations [29].

The nature and severity of the impairment has an important influence on participation. Not only in the participation areas family of origin and intimate relationships but also in the other areas, in the overall study [27] we saw that different impairments may pose different participation barriers and facilitators. Persons with physical impairment mainly reported mobility barriers, be it in the area of work, housing, education, or leisure time. Further, barriers were mentioned in the area of intimate relationships, whereas contacts to friends and family did not pose as many problems. Persons with cognitive impairment were mostly living in residential care settings or in the family of origin. They predominantly had social contact with other people with cognitive impairment and their family of origin. Often a major source of help, the family of origin also limited participation, self-determined decision-making, and opportunities for sexuality and intimate relationships. Persons with psychiatric impairment often reported problems with their family of origin (conflicts, loss of contact). Also due to their psychiatric disorders (e.g., anxiety disorder, depression) and self-experienced societal stigmatization, they often lived isolated and very secluded.

The results of the present study in the area ‘family of origin’ can be summarized as follows: They indicate that good relations with and integration in the family of origin is not only important for participation in the family of origin but also for participation in other areas of life (housing, work, relationships, etc.), as already demonstrated [14, 30, 31]. And, vice versa, financial resources and other areas of life such as housing impact participation within family of origin. But relations within family of origin are not easy to handle: Conflicts arise, and some persons with an impairment do not feel understood by their family. Ties to the family of origin are sometimes completely broken off, especially for persons with psychiatric impairment. Some interviewees, especially persons with a cognitive impairment, had to deal with overprotective parenting that limited participation and self-determined decision-making. There is a lack today of extensive studies on the role of the family of origin as an important barrier to the participation of adults with impairment(s). Further research is needed, especially longitudinal studies that accompany families and children from early childhood to emerging adulthood to better understand and validate familial strategies that facilitate or hinder participation of the person with an impairment. But based on the present study, we can already derive implications for practitioners on how to facilitate participation in the family of origin. Families should be supported and offered professional services from an early age on (e.g., provided by social workers, special needs experts). For families with children with cognitive impairment, a focus should be put on helping these families successfully detach and restructure relationships in the trajectory from childhood to adulthood and create the best possible participation possibilities. Depending on early or late onset of a psychiatric impairment, this might be also the case for families with children/persons with psychiatric impairment. But in these families—drawing on our results—it seems important first of all that health professionals and social workers provide assistance in maintaining intra-family relationships, and that they sensitize family members to mental health issues and their potential impacts on family life and intra-familial conflicts. Further, advocacy for and self-advocacy of persons with impairment should be strengthened (see more detail below).

Let us now put the results on the participation area ‘intimate relationships’ into the context of existing research and formulate potential implications for practitioners. Other studies have already identified barriers when it comes to intimate relationships of persons with cognitive or physical impairments [17, 32]. The influence of parents on the sexuality of adult children with cognitive impairment was briefly mentioned in a qualitative Swiss study [32]. Despite longing for a relationship, a lot of people with impairments live as single persons. Our results confirm and deepen previous results. We saw that especially persons with cognitive or physical impairments face barriers. Sexuality and sexual needs are tabooed by the environment around people with impairments and by society as a whole. The voices of persons with impairments—especially those with cognitive or physical impairments—tell us that the societal trope of constructing people with impairments as non-sexual subjects limits participation in intimate relationships. Sexuality and family planning—especially in people with cognitive impairment—are sometimes severely restricted, as stated also in the report by the Swiss umbrella association Inclusion Handicap to the United Nations [29]. The coping strategies for dealing with barriers are very diverse. They range from functional strategies, such as seeking support in getting to know an intimate partner, to non-functional strategies, such as self-stigmatization and complete withdrawal from the public space. There is also an urgent need in Switzerland to further sensitize families, parents, and society overall to the sexual and intimate needs of people with impairments. Moreover, our study participants called for more structural opportunities and places to get to know potential partners, such as singles events and meetups. And the potential of social media and the Internet as a partner market for persons with impairments should be explored [32]. Further, personal assistants of persons with physical impairment should be trained adequately in how to facilitate sexual intimacy within the partnership of a person with physical impairment. Parents with children with cognitive impairment should receive information, advice and support early on about sexual needs of their children in the transition to adulthood and about how to support these needs best. In order to develop evidence-based, tailored professional measures for these families (e.g., provided by social workers or special needs specialists), also in the participation area of intimate relationships, further research should be conducted with a longitudinal perspective (from childhood to emerging adulthood). Moreover, also in this area of participation, advocacy for people with impairments should be increased—advocacy not only by society, social workers, special needs experts and health professionals but also by strengthening the resources of individuals and groups of people with impairments for self-advocacy. Both in the relationship with their own family of origin and in intimate relationships, we saw clearly that when people can stand up for themselves, speak out and speak up for their rights—practice self-advocacy—this has a positive effect on their opportunities for participation. This means concretely that associations for people with impairments should create spaces in which individuals with impairments can empower themselves, take over control, and develop skills in empowerment. In particular, people with cognitive impairment should be given more capacity and power in defending their (sexual) rights, especially when their own parents—as our data identified—sometimes curtail sexual rights. There is evidence that membership in self-advocacy groups has positive effects and furthers participation and social inclusion of people with intellectual disability also when it comes to social connections and relationships [33, 34].

After mentioning implications for the areas ‘family of origin’ and ‘intimate relationships’ we now formulate overall implications for practitioners and policy: Due to the wide spectrum of the identified participation continuum, barriers in all areas and in both settings—separative and inclusion-oriented—have to be torn down, and the transitions to inclusive settings have to be improved. Financial scarcity is a major barrier to participation in all areas and settings [27]. Hence, financial resources of persons with impairment should be strengthened and better secured by the Swiss state. Financial restrictions in disability insurance pensions on people with disabilities, especially those with psychiatric impairment, have increased in recent years. Also in other areas, for example in providing adequate housing, the Swiss system for managing and allocating financial resources for persons with impairments, especially for those with cognitive impairment, is not yet set up ideally. In this system and current practices, residential care is given preference over self-determined independent living (own/rented apartment) and assisted living [29, 35]. There is too little freedom of choice. System and practices should be changed and become more flexible to better support self-determined participation and social inclusion of people with impairments. This would also facilitate participation in the areas of family of origin and intimate relationships. The CRPD, ratified by Switzerland, is an important means to advocate for people with disability and to boost social inclusion for these persons. Advocacy should be strengthened on all levels. A good example on the societal (macro) and meso level is the report to the United Nations by Inclusion Handicap, the Swiss umbrella association of disability organizations [29]. But, as already mentioned, we need also strong initiatives to booster self-advocacy, so that people with impairments, especially those with cognitive impairment, are better empowered to speak up.

### Limitations

Tests for data saturation provided evidence that a good saturation was reached overall. At the end of the study, already established code axes and concepts were confirmed by new interview data. However, data saturation and the range of the study must be considered in light of the study’s main objective. In a holistic way, we reconstructed the phenomenon of participation across various forms of impairment and areas of life. This was against the background of the subjective experience and assessment of participation by people with impairments. We identified differences in participation between persons with different impairments. But researching these differences was not at the focus of our study. The sample size for each subgroup of impairment (cognitive, physical vs. psychiatric) was quite small. Therefore, differences identified between people with cognitive, physical, or psychiatric impairments need to be further developed and validated with additional data. Another limitation is that persons with very high support needs and severe sensory impairments could not be included in this study due to the methodical approach (problem-centered interviewing). Therefore, the empirically developed grounded theory on participation and the identified barriers and facilitators in the family of origin and in intimate relationships need to be further developed and validated with additional data. In general, it would be a gain for disability studies and public health research to put the hereby-presented grounded theory on participation as a continuum in an international context so as to further validate and elaborate the theory in different countries, different areas of participation, and with people with various impairments. Hereby, future research should also increasingly survey parents and professionals in order to validate and better understand the barriers identified by the interviewees. The barriers revealed in this study are framed in the subjective perspective of persons with impairment. To fully understand participation restrictions, their subjective views should be compared and contrasted with the views of the professional and family environments. This approach would also help provide answers to the following questions that our findings raise: When and for what reason do professionals (social workers, caregivers) or relatives hinder the transition to more inclusive settings? At what moments and why do professionals exert pressure to switch to more inclusive settings, despite a person’s desire to remain in a separative setting?

## Conclusions

This grounded theory study provides insights into successful and restricted participation in the German-speaking part of Switzerland from the subjective perspective of adults with cognitive, physical, and psychiatric impairments. Various barriers were identified in both separative and inclusion-oriented settings. Moreover, many people cannot switch to more inclusive settings. Therefore, central principles in the CRPD such as “full and effective participation and inclusion in society” are not met in Switzerland. People with impairments are still prevented from living out their sexuality and love relationships freely and self-determinedly. Families are not only supportive but also in some cases, especially with persons with cognitive impairment, hinder their adult children’s self-determination. The results of our study can instruct policy and practice by pinpointing that participation barriers in all settings should be torn down and trajectories to inclusion-oriented settings should be strengthened. For example, families with children with cognitive impairment(s) should be supported from early on to create the best possible participation possibilities for the (adult) person with impairment(s) and to support the family of origin itself. In Switzerland, there is also an urgent need to further sensitize families, parents, professionals, and society overall to the sexual and intimate needs and sexual rights of persons with impairments. This is especially the case for persons with cognitive or physical impairments. Further, the financial security of people with impairments, advocacy for people with impairments, and self-advocacy of people with impairments should be strengthened. Drawing on the paradigm of inclusion, the main burden of change and adaptation always lies with society, institutions, and systems and not with the individual with an impairment [36].

## Supplementary information

**Additional file 1.** Completed COREQ checklist for the manuscript.

## Data Availability

The verbal data generated for and analyzed during the current study are not publicly available due to the privacy and the protection of the participants. Even though the interviews have been fully anonymized (names, places, etc.), the participants’ narratives could lead to individual identification by close family, partners, friends, etc.

## Abbreviations

ICF: International Classification of Functioning, Disability and Health
CRPD: Convention on the Rights of Persons with Disabilities
WHO: World Health Organization
