# Supplementary material for: Participation of adults with cognitive, physical, or psychiatric impairments in family of origin and intimate relationships: a grounded theory study
Source: BMC Public Health. 2020 May 7;20:642. doi: 10.1186/s12889-020-08770-x (PMC7206802; doi:10.1186/s12889-020-08770-x)
Supplement: Supplementary file 1 — Additional file 1. Completed COREQ checklist for the manuscript. [file 12889_2020_8770_MOESM1_ESM.docx]

**Completed** [**COREQ**](https://academic.oup.com/intqhc/article/19/6/349/1791966) **checklist for the manuscript**

**Participation of adults with cognitive, physical, or psychiatric impairments in family of origin and intimate relationships: a grounded theory study**

Andreas Pfister, Pia Georgi-Tscherry, Fabian Berger, and Michaela Studer

| **No** | **Item** | **Guide questions / description** | **Response** |
| --- | --- | --- | --- |
| Domain 1: research team and reflexivity | | | |
| 1 | Interviewer/facilitator | Which author/s conducted the interview or focus group? | Author’s contribution, p. 32 |
| 2 | Credentials | What were the researcher’s credentials? E.g. PhD, MD | Title page, p. 1 |
| 3 | Occupation | What was their occupation at the time of the study? | Title page, p. 1 |
| 4 | Gender | Was the researcher male or female? | Title page, p. 1 AND Author’s contribution, p. 32 |
| 5 | Experience and training | What experience or training did the researcher have? | Title page, p. 1 |
| 6 | Relationship established | Was a relationship established prior to study commencement? | Methods, Paragraph ‘Recruitment of participants’, pp. 7-8 |
| 7 | Participant knowledge of the  interviewer | What did the participants know about the researcher? e.g. personal goals, reasons for doing the research | Methods, Paragraph ‘Problem-centred interviewing’, p. 8 |
| 8 | Interviewer characteristics | What characteristics were reported about the interviewer/facilitator? e.g. Bias, assumptions, reasons and interests in the research topic | N/A |
| Domain 2: study design | | | |
| 9 | Methodological orientation and  Theory | What methodological orientation was stated to underpin the study? e.g. grounded theory, discourse analysis, ethnography, phenomenology, content analysis | Methods, Paragraph 1+2, p. 6 |
| 10 | Sample | How were participants selected? e.g. purposive, convenience, consecutive, snowball | Methods, Paragraphs ‘Inclusion criteria and sampling strategy’ AND ‘Recruitment of participants’, pp. 6-8 |
| 11 | Method of approach | How were participants approached? e.g. face-to-face, telephone, mail, email | Methods, Paragraph ‘Recruitment of participants’, pp. 7-8 |
| 12 | Sample size | How many participants were in the study? | Methods, Paragraph ‘Participant characteristics’, p. 9 (including Table 1, p. 35) |
| 13 | Non-participation | How many people refused to participate or dropped out? Reasons? | N/A |
| 14 | Setting of data collection | Where was the data collected? e.g. home, clinic, workplace | Methods, Paragraph ‘Problem-centred interviewing’, p. 8 |
| 15 | Presence of non-participants | Was anyone else present besides the participants and researchers? | No! N/A |
| 16 | Description of sample | What are the important characteristics of the sample? e.g. demographic data, date | Methods, Data collection (Paragraph 1, p. 6) AND Paragraph ‘Participant characteristics’, p. 9 (including Table 1, p. 35) |
| 17 | Interview guide | Were questions, prompts, guides provided by the authors? Was it pilot tested? | Methods, Paragraph ‘Problem-centred interviewing’, pp. 8-9 |
| 18 | Repeat interviews | Were repeat interviews carried out? If yes, how many? | No! N/A |
| 19 | Audio/visual recording | Audio/visual recording Did the research use audio or visual recording to collect the data? | Methods, Paragraph ‘Problem-centred interviewing’, p. 8 |
| 20 | Field notes | Were field notes made during and/or after the interview or focus group? | Methods, Paragraph ‘Problem-centred interviewing’, p. 9 |
| 21 | Duration | What was the duration of the interviews or focus group? | Methods, Paragraph ‘Problem-centred interviewing’, p. 8 |
| 22 | Data saturation | Was data saturation discussed? | Discussion, Paragraph ‘Limitations’, p. 28-29 |
| 23 | Transcripts returned | Were transcripts returned to participants for comment and/or correction? | No! Audio files were fully transcribed! N/A |
| Domain 3: analysis and findings | | | |
| 24 | Number of data coders | Number of data coders How many data coders coded the data? | Methods, Paragraph ‘Data analysis’, pp. 9-10 |
| 25 | Description of the coding tree | Did authors provide a description of the coding tree? | N/A due to grounded theory methodology |
| 26 | Derivation of themes | Were themes identified in advance or derived from the data? | Methods, Paragraph ‘Data analysis’, pp. 9-10 |
| 27 | Software | What software, if applicable, was used to manage the data? | Methods, Paragraph ‘Data analysis’, pp. 9 |
| 28 | Participant checking | Did participants provide feedback on the findings? | Methods, Paragraph ‘Data analysis’, pp. 10 |
| 29 | Quotations presented | Were participant quotations presented to illustrate the themes / findings? Was each quotation identified? e.g. participant number | Results, all paragraphs and quotations, pp. 10-23 |
| 30 | Data and findings consistent | Was there consistency between the data presented and the findings? | Results, all paragraphs, pp. 10-23 |
| 31 | Clarity of major themes | Clarity of major themes Were major themes clearly presented in the findings? | Results, all paragraphs, pp. 10-23 |
| 32 | Clarity of minor themes | Clarity of minor themes Is there a description of diverse cases or discussion of minor themes? | Results, all paragraphs, pp. 10-23 AND Discussion, all paragraphs, pp. 23-28 |

31 March 2020

Lucerne, Andreas Pfister
